# Large intestinal nutritional and physicochemical parameters from different dog sizes reshape canine microbiota structure and functions *in vitro*

**DOI:** 10.1080/21655979.2024.2325713

**Published:** 2024-03-12

**Authors:** Charlotte Deschamps, Delphine Humbert, Sandrine Chalancon, Caroline Achard, Emmanuelle Apper, Sylvain Denis, Stéphanie Blanquet-Diot

**Affiliations:** a Université Clermont Auvergne, UMR 454 MEDIS UCA-INRAE, Clermont-Ferrand, Puy-de-Dôme, France; b Lallemand Animal Nutrition, Blagnac Cedex, Haute-Garonne, France; c Dômes Pharma, Pont-du-Château, Puy-de-Dôme, France

**Keywords:** Dog, gut microbiota, *in vitro gut model*, CANIM-ARCOL, mucus

## Abstract

Different dog sizes are associated with variations in large intestinal physiology including gut microbiota, which plays a key role in animal health. This study aims to evaluate, using the CANIM-ARCOL (Canine Mucosal Artificial Colon), the relative importance of gut microbes *versus* physicochemical and nutritional parameters of the canine colonic environment in shaping microbiota structure and functions. CANIM-ARCOL was set up to reproduce nutrient availability, bile acid profiles, colonic pH, and transit time from small, medium, or large dogs according to *in vivo* data, while bioreactors were all inoculated with a fecal sample collected from medium size dogs (*n* = 2). Applying different dog size parameters resulted in a positive association between size and gas or SCFA production, as well as distinct microbiota profiles as revealed by 16S Metabarcoding. Comparisons with *in vivo* data from canine stools and previous *in vitro* results obtained when CANIM-ARCOL was inoculated with fecal samples from three dog sizes revealed that environmental colonic parameters were sufficient to drive microbiota functions. However, size-related fecal microbes were necessary to accurately reproduce *in vitro* the colonic ecosystem of small, medium, and large dogs. For the first time, this study provides mechanistic insights on which parameters from colonic ecosystem mainly drive canine microbiota in relation to dog size. The CANIM-ARCOL can be used as a relevant *in vitro* platform to unravel interactions between food or pharma compounds and canine colonic microbiota, under different dog size conditions. The potential of the model will be extended soon to diseased situations (e.g. chronic enteropathies or obesity).

## Highlights

- Environmental colonic parameters (such as nutrient availability, transit time, or pH) were sufficient to drive microbiota at the functional level in the CANIM-ARCOL *in vitro* gut model.
- Size-related fecal microbes were necessary to accurately reproduce the colonic environment of small, medium, and large dogs.
- CANIM-ARCOL model can be used as a relevant in vitro tool to decipher the relative importance of microbiota versus environmental colonic parameters in food and pharma studies.

## Introduction

It is now acknowledged that gut microbiota plays a crucial role in the health of mammalian hosts, including dogs. Each compartment of the canine gastrointestinal tract is colonized by a resident microbiota mainly composed by bacteria, viruses, fungi, and protozoa, with the higher diversity and abundance found in the large intestine [1,2]. Even if evidences remain scarce in dogs [3–5], it can be assumed, based on the human situation, that canine microbiota varies from the digestive lumen to the intestinal epithelium surface, where a mucus layer provides nutrients and habitat for specific microbes. The intestinal microbiome protects against pathogens, educates the host immune system, and has important metabolic functions, such as food digestion leading to major end-fermentation products like short-chain fatty acid (SCFA), ammonia and gases, and bile acid metabolism [6,7].

Up to now, there are few data in dogs investigating how canine body weight can impact digestive physiology and gut microbiota [8]. Most of recognized changes in gastrointestinal physiology are related to the large intestine, with a decrease in colonic pH together with increase in transit time and permeability associated to dog size [9–14]. Regarding the effect of canine body weight on gut microbiota composition and activities, the only available data are provided from stool analysis. Those studies seem to indicate a positive correlation between body weight and carbohydrate/protein fermentation capacity [15–20], resulting in higher SCFA and ammonia concentrations in fecal samples from large dogs [11]. Fecal bile acid profiles are also impacted by dog size, with an apparent decrease of total bile acids, as well as primary to secondary bile acid ratios, when body weight increases [21]. Lastly, data from dog stools suggest that relative abundances of *Proteobacteria* and *Actinobacteria* decreased with body weight [8]. However, based on the few available studies, canine colonic and fecal microbiotas differ in terms of bacterial composition and diversity [2,22].

Given the paucity of data related to dog size effect on large intestinal microbiota structure and functions in dogs, an alternative option is to use *in vitro* models of the canine colon to answer such question. This alternative strategy is fully in line with European ‘3 R’ rules and provides cost, technical, ethical, and regulatory benefits compared to *in vivo* assays [23,24]. Two dynamic models of the canine colon have been recently developed, namely the Mucosal Simulator of the Canine Intestinal Microbial Environment (M-SCIME) [25] and the Canine Mucosal Artificial Colon (CANIM-ARCOL) [26]. Only the second one has been adapted to simulate the specific conditions (i.e. pH, transit time, nutrients, bile acid profiles) found in the colon of three dog sizes (i.e. small under 10 kg, medium from 10 to 30 kg and large size over 30 kg), with great *in vivo-in vitro* correlations. In those studies, *in vitro* colon models have been inoculated with fecal samples from size-related colonic conditions (e.g. medium size bioreactors were inoculated with stool from medium size dogs). It would be now of great interest to investigate if colonic nutritional and physicochemical parameters from different dog sizes are sufficient to reshape microbiota profiles *in vitro*.

In this context, we performed *in vitro* fermentations in the CANIM-ARCOL inoculated with medium dog size stools but set up to reproduce small, medium, or large colonic conditions. Samples were regularly collected in bioreactors to analyze the composition of both lumen and mucus-associated microbiota and monitor gut microbes’ activities through SCFA, gas, and ammonia measurement.

## Materials and methods

### Fecal samples collection and treatment

Two healthy dogs from medium size were used as stool donors for *in vitro* experiments (Table 1): a female Labrador (dog A, 25 kg) and a male Samoyed (dog B, 22.5 kg). Both dogs were owner-pets, fed with commercial dry food, with access to outdoor. Immediately after defecation, fecal samples were transferred into a sterile recipient, placed in an airtight anaerobic box (GENbag anaer gas pack systems, Biomerieux, France), transported and processed at the laboratory within 3 h. In an anaerobic chamber (COY laboratories, Grass Lake, USA), stool samples were manually homogenized, and 3.75 g of feces were resuspended in 100 mL of 30 mM sterile sodium phosphate buffer (pH 6.0), mixed and filtered (500 µm inox sieve).

**Table 1.** Characteristics of healthy adult dogs from medium size used as fecal donors for *in vitro* experiments. M: male, F: female, BCS: body condition score (ranging from 1 – very thin to 5 – obese, 3 corresponding to ideal weight).

Size	Dog_id	Breed	Sex	Sterilization	Age (years)	BCS	Weight (kg)	Garden access	Feed
Medium	A	Labrador	F	Yes	9	4	25	Yes	Dry
	B	Samoyed	M	Yes	2.5	3	22.5	Yes	Dry

### Description and set-up of the CANIM-ARCOL model

**Figure 1.**
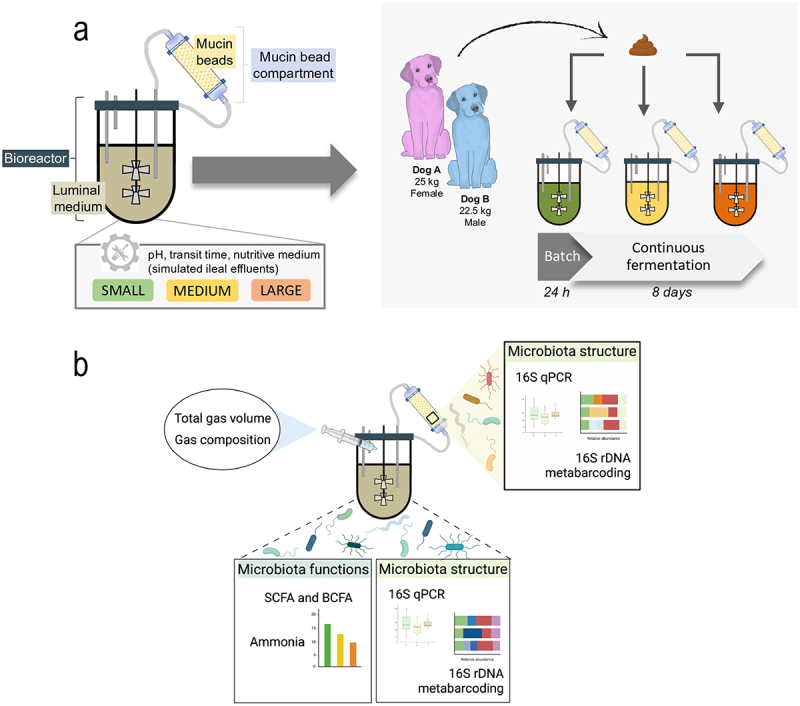
Experimental design in the CANIM-ARCOL. (a) The CANIM-ARCOL was inoculated with fecal samples from two medium dogs (one female and one male, i.e. two biological replicates). Three bioreactors corresponding to three sizes conditions (i.e. small under 10 kg, medium from 10 to 30 kg and large over 30 kg) were run in parallel for 9 days. (b) Samples were regularly collected in the atmospheric phase, in the luminal medium and from mucin beads to monitor microbiota composition and fermentation metabolites.

CANIM-ARCOL is a one-stage fermentation system (MiniBio, Applikon, Delft, The Netherlands), inoculated with stool samples and used under continuous conditions to simulate the nutritional, physicochemical, and microbial conditions found in the large intestine of dogs, as previously described in [26]. Briefly, the *in vitro* model is composed of a main bioreactor simulating the colonic luminal medium and an airtight glass vessel connected to this bioreactor and containing mucin beads to reproduce the mucosal compartment (Figure 1a). At the beginning of experiments, 100 mL of fecal suspension from each dog was added per bioreactor to 200 mL of sterile canine-adapted nutritive medium simulating the composition of ileal effluents (Table 2). To ensure anaerobic condition at the beginning of fermentation, the bioreactor was operated with an initial sparging with O_2_-free N_2_ gas. Afterward, during the fermentation course, anaerobic condition was maintained by the sole activity of resident microbiota. The *in vitro* model was kept at canine body temperature (i.e. 39°C). pH and redox potential were constantly recorded (Applikon, The Netherlands) and pH was adjusted to the setpoint values with 2 M NaOH. The nutritive medium was continuously introduced into the main bioreactor, while the fermentation medium was automatically withdrawn, ensuring the appropriate colonic retention time. Every 2 days, mucin beads from the mucosal compartment were renewed by fresh sterile ones under a constant flow of CO_2_ to avoid oxygen entrance, as previously described [27]. In the present study, the CANIM-ARCOL was set up with nutritional and physicochemical parameters adapted to three dog sizes as previously validated and detailed in Table 2 [26].

**Table 2.** Nutritional and physicochemical parameters used to set up the CANIM-ARCOL under three dog size conditions. rpm: rotation per minute.

Size	Small	Medium	Large
Weight (kg)	**<10**	**10–30**	**>30**
**Bioreactor’s parameters**			
Temperature	39°C	39°C	39°C
Residence time	5 h	9 h	15 h
pH	6.6	6.5	6.2
Stirring	400 rpm	400 rpm	400 rpm
**Nutritive medium composition (in g/L)**			
Proteins	17.2	27.0	35.6
Carbohydrates	0.9	1.3	1.8
Lipids	1.6	2.4	3.2
Fibers	3.3	5.2	6.8
**Bile acids composition in the nutritive medium (mg/L)**			
Cholic acid	108 mg 35%	55 mg 10%	32 mg 5%
Chenodeoxycholic acid	31 mg 10%	27 mg 5%	32 mg 5%
Deoxycholic acid	124 mg 40%	327 mg 60%	418 mg 65%
Lithocholic acid	46 mg 15%	136 mg 25%	161 mg 25%

### Experimental design and sampling

For each experiment, three bioreactors were inoculated with a same fecal sample from a medium size dog and run in parallel (Figure 1a). Each bioreactor was set up with parameters corresponding to one of the three size conditions (i.e. small, medium, or large dog sizes), based on *in vivo* data as previously reviewed [8]. Fermentations were run under batch conditions for 24 h and then under continuous conditions for 8 additional days. Samples were collected daily (Figure 1b) in the fermentation medium (luminal medium) for further analysis of microbiota composition (qPCR and 16S Metabarcoding) and gut microbial activities through SCFA and ammonia measurement. Every 2 days, mucin beads were collected for analysis of mucus-associated microbiota (qPCR and 16S Metabarcoding). Mucin beads were washed twice in sterile sodium phosphate buffer and stored at −80°C before downstream analysis. The atmospheric phase was also sampled every day to follow anaerobiosis and determine gas composition and production (total volume of gas) thanks to a sampling bag connected to the condenser (Figure 1b).

### DNA extraction

Genomic DNA was extracted from luminal medium samples and mucin beads using the QIAamp Fast DNA Stool Mini Kit (Qiagen, Germany) following manufacturer’s instructions with the following modifications. Prior to DNA extraction, luminal samples were centrifuged (18 000 rcf, 15 min, 4°C), and the pellets were collected. Pellets and mucin bead samples were then incubated 10 min with sterile citrate buffer (sodium citrate 55 mM and NaCl 154 mM) at 37°C, before vortexing (maximal speed, 15 sec) and centrifuging again (8000 rcf, 1 min). Then, a step of mechanical disruption using a bead beater (5 min, 20 beat/s) was made with 300 mg sterile glass beads (diameter ranging from 100 to 600 µm). DNA quantity was evaluated using the Qubit dsDNA Broad Range Assay Kit (Invitrogen, USA) with a Qubit 3.0 Fluorometer (Invitrogen, USA). Samples were stored at −20°C prior to microbiota analysis.

### Quantitative PCR

Total bacteria were quantified by qPCR using primers described in Table 3. Real-time PCR assays were performed in a Biorad CFX96TM Real-Time System (Bio-Rad Laboratories, USA) using Takyon Low ROX SYBR 2X MasterMix blue dTTP kit (Eurogentec, Belgium). Each reaction was run in duplicate in a final volume of 10 μL with 5 μL of MasterMix, 0.45 μL of each primer (10 μM), 1 μL of DNA sample, and 3.1 μL of ultra-pure water. Amplifications were carried out using the following ramping profile: 1 cycle at 95°C for 5 min, followed by 40 cycles of 95°C for 30 s, 58°C for 30 sec. A melting step was added to ensure primer specificity. Standard curve was generated from 10-fold dilutions of bacterial DNA (isolated from a pure culture of bacteria), allowing the calculation of DNA concentrations from extracted samples.

**Table 3.** Primers used for qPCR and 16S metabarcoding analysis.

Primer name	Sequence 5′-3′	Target	Annealing temperature (°C)	References
**qPCR primers**				
BAC338R BAC516F	ACTCCTACGGGAGGCAG GTATTACCGCGGCTGCTG	Total bacteria	58	Yu et al. [28]
**Metabarcoding primers**				
V3-341F V4-806 R	CCTACGGGAGGCAGCAG GGACTACNVGGGTWTCTAAT	Bacteria	55	Beaumont et al. (58)

### 16S metabarcoding and data analysis

Bacterial V3-V4 regions of 16S ribosomal DNA (rRNA) were amplified using primers described in Table 3. High-throughput sequencing was performed on an Illumina MiSeq sequencer by the GeT-PlaGe core facility (INRAe Transfer, Toulouse, France). MiSeq Reagent Kit v3 was used according to the manufacturer’s instruction (Illumina Inc., San Diego, CA). Bioinformatic analysis was performed using R studio software and using rANOMALY package [29]. Prior to analysis, raw data were demultiplexed and quality filtered using DADA2 R-package [30]. Reads with quality score under 2 were truncated. Reads under 100 pb length were removed as well as sequences similar to PhiX DNA used as a spike-in control for MiSeq runs. Filtered sequences were dereplicated and cleaned for chimeras (DADA2). Taxonomic classification of the sequences was then performed with DECIPHER package [31]. Assignations from both SILVA 138 release [32] and GTDB_bac120_arc122 [33] databases were merged using the assign_taxo_fun function from rANOMALY R-package based on IDTAXA, with a 60% confidence cutoff [29]. A phylogenetic tree was then reconstructed using DECIPHER.

### Gas analysis

Analysis of O_2_, N_2_, CO_2_, CH_4_, and H_2_ gas produced during the fermentation process was performed using 490 micro-gas chromatography (Agilent Technologies, USA) coupled with a micro-TCD detector (Agilent Technologies, USA). Molecular Sieve 5A and Porapak Q (Agilent Technologies, USA) series columns were used. Gas composition was determined using calibration curves made from ambient air (78.09% N_2_, 20.95% O_2_, 0.04% CO_2_) and three gas mixtures A (5% CO_2_, 5% H_2_, 90% N_2_), B (20% CO_2_, 80% H_2_), and C (20% CO_2_, 20% CH_4_, 20% H_2_, 40% N_2_). Technical replicates were performed for each sample, and results were expressed as relative percentages.

### Short-chain fatty acid analysis

For SCFA analysis, 1.5 mL of luminal medium samples were centrifuged (18 000 rcf, 15 min, 4°C), and 900 μL of supernatant was diluted at 1/10 into H_2_SO_4_ 0.04 M mobile phase, vortexed, and filtered (0.22 μm). The three major SCFAs (acetate, propionate, and butyrate) were quantified by high-performance liquid chromatography (HPLC) (Elite LaChrom, Merck HITACHI, USA) coupled with a DAD diode. The HPLC column (Concise Separations, ICE-99-9865) and its guard column were maintained at 50°C. Sulfuric acid 0.04 M was used as mobile phase and SCFA were separated at a flow rate of 0.6 mL/min. Data were obtained and analyzed by the EZChrom Elite software at 205 nm. SCFA concentrations were calculated from calibration curves established from known concentration solutions of acetate, propionate, and butyrate (0, 10, 25, and 40 mM) and data expressed as mM or relative percentages.

### Ammonia quantification

Total ammonia was measured using the Ammonia assay kit (LIBIOS, France) following manufacturer’s instructions. Results were expressed in mmol/L.

### Statistical analysis

Statistical analyses on microbiota activity (gas, SCFA, ammonia) and α-diversity indexes (number of observed ASVs and Shannon index) from metabarcoding data were processed using GraphPad Prism software version 9.4.1 (GraphPad Software, USA). Data normal distribution was verified by combining Anderson-Darling, D’Agostino & Pearson, Shapiro–Wilk, and Kolmogorov–Smirnov tests, and homoscedasticity was checked using the Fisher test. Then, appropriate statistical analysis was applied (either one-way ANOVA, *t*-test, Mann–Whitney, or Welch’s tests). First, principal coordinate analysis (PCoA, data not shown) was performed followed by Non-Metric Multidimensional Scaling (NMDS), highlighting important size and microenvironment (i.e. luminal medium and mucin beads) effects. Constraint redundancy analysis (RDA) was then performed with age, weight, sex, size, microenvironment, donor, and time as variables of the model, first with all parameters and then with removal of either size or microenvironment variables. Bray Curtis distances were used for each analysis, and significance between groups was assessed with a one- or two-way ANOVA. Discriminant analyses (sPLS-DA) were finally performed using MixOmics package [34]. Pearson correlations between physicochemical or nutritional variables and bacterial families were established using the ‘microeco’ R-package [35].

## Results

### Faecal inoculum characterization

**Figure 2.**
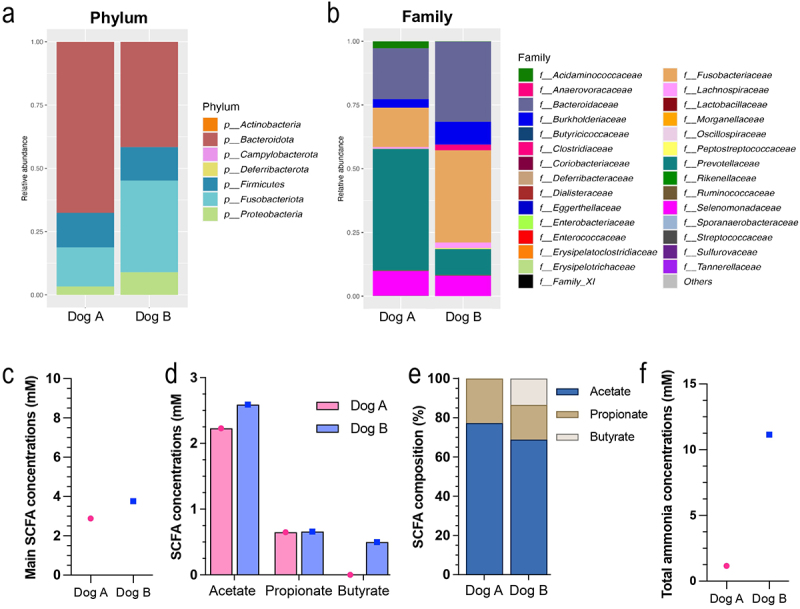
Stool characterization for each canine donor. Stool samples were collected from two healthy medium dogs. Microbiota composition was analyzed by 16S metabarcoding. Bacterial abundances are represented at the phylum (a) family (b) levels. The three main SCFA (i.e. acetate, propionate and butyrate) were measured and expressed as total concentration (c and d) and relative percentages (e). Ammonia concentrations are given in (f).

Stools used for bioreactor inoculation were characterized (Figure 2). Alpha-diversity was similar between the two fecal samples with a Shannon index of 2.99 for dog A and 2.98 for dog B. Fecal bacterial profiles at the phylum level (Figure 2a) were similar between the two donors with a majority of *Bacteroidota* (67% in dog A and 42% in dog B), followed by *Fusobacteriota* (15% in dog A, 36% in dog B), *Firmicutes* (13%), and *Proteobacteria* (3% in dog A, 9% in dog B). At the family level (Figure 2b), dog B showed higher abundance in *Bacteroidaceae, Burkholderiaceae, Clostridiaceae*, and *Fusobacteriaceae* families compared to dog A, whereas *Prevotellaceae* was less abundant. Even if total fecal SCFA concentration (Figure 2c) was similar between the two dogs, different SCFA profiles (Figures 2d,e) were observed with a majority of acetate in both donors, but no butyrate in dog A. Similarly, ammonia concentrations differed between the two dogs with a 10-fold higher concentration in dog B (Figure 2f).

### Impact of size conditions on canine colonic microbiota structure

**Figure 3.**
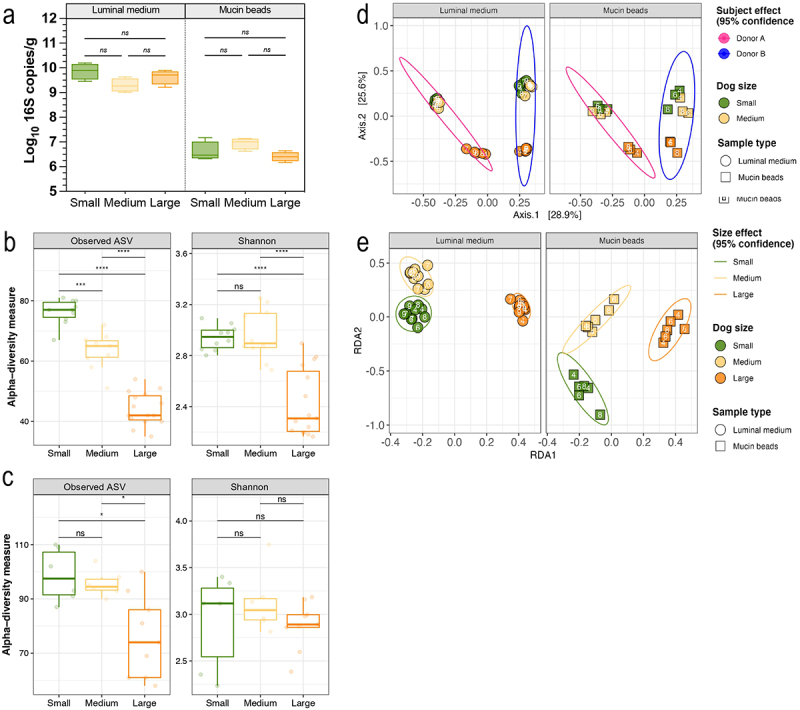
Impact of dog size on bacterial load and microbial diversity in the CANIM-ARCOL. Fermentations were performed in the CANIM-ARCOL under three dog size conditions (i.e. small, medium, large), after inoculation with stools from medium size dogs (*n* = 2). Total bacteria were quantified and expressed as Log_10_ 16S copies/g (a). Lumen and mucus-associated microbiota composition was analyzed by 16S metabarcoding and diversity indexes were calculated based on ASV table. α-diversity indexes (observed ASVs and Shannon) calculated from days 4 to 9 are represented as box plots in the luminal medium (b) and mucin beads (c). Beta-diversity of samples from days 4 to 9 was analyzed by PCoA showing clear donor (d) effect. Redundancy analysis (RDA) without donor effect based on Bray–Curtis distances indicated strong size effect (e). Significant differences based on Kruskal and Wallis test are presented as * *p* < 0.05, *** *p* < 0.001, **** *p* < 0.0001. ns = non-significant difference (*p* > 0.05).

CANIM-ARCOL was used to run colonic fermentations using small, medium, or large size parameters while inoculated only with a medium stool. Total bacteria levels (Figure 3a) were similar whatever the size condition, but with higher amounts in the luminal medium (10 Log_10_ 16S copies/g) than in mucin beads (6 Log_10_ 16S copies/g). In the luminal medium (Figure 3b), number of observed ASV was negatively correlated with breed format (*p* < 0.001), while only Shannon index from large size condition was significantly lower than the one obtained for small and medium (*p* < 0.0001). In the mucin beads (Figure 3c), number of observed ASV was significantly lower for large condition (*p* < 0.05), whereas Shannon index was not different. Whatever the size condition, alpha diversity was higher in mucin beads compared to luminal medium. Principal component analysis (PCoA) based on ASV composition and Bray-Curtis distance showed strong effects of stool donor, in both colonic microenvironments (Figure 3d). Redundancy analysis (RDA) removing donor effect demonstrated a clear (*p* < 0.0001) clustering by size, again in both the luminal medium and mucin beads (Figure 3e). In addition, samples associated to large condition clustered apart from small and medium groups.

**Figure 4.**
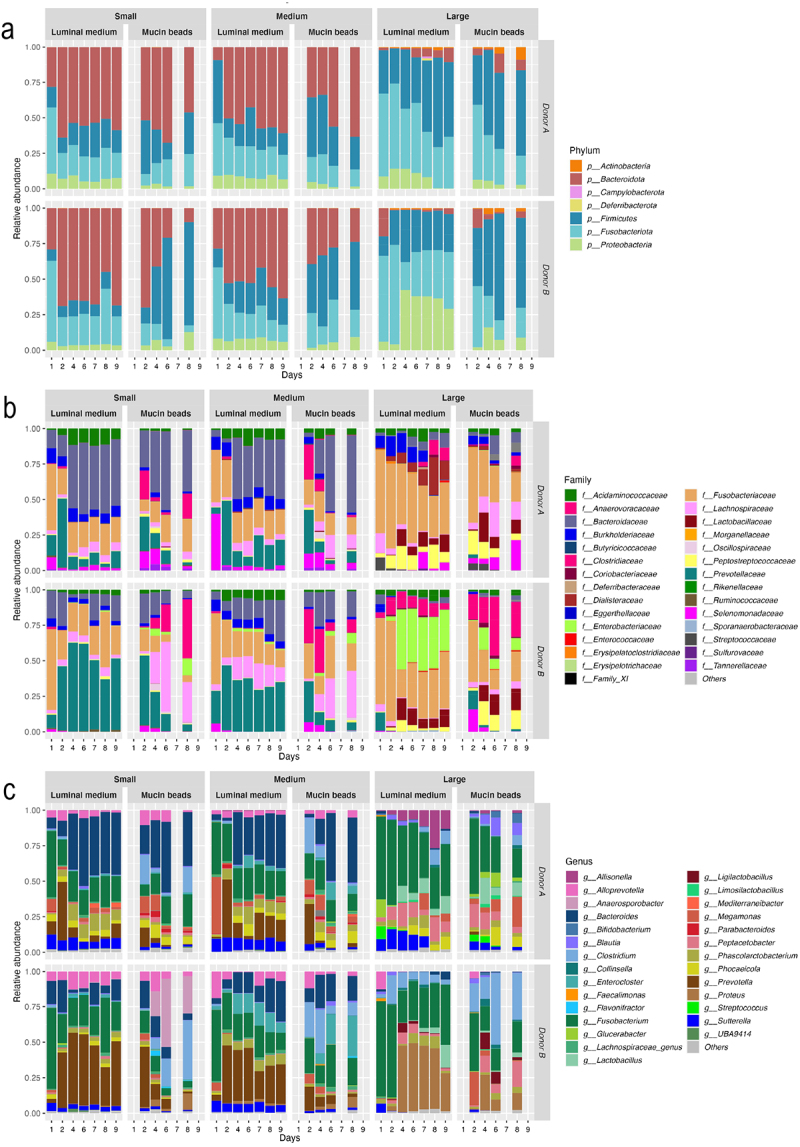
Impact of dog size on microbial composition in the CANIM-ARCOL. Fermentations were performed in the CANIM-ARCOL under three dog size conditions (i.e. small, medium, large), after inoculation with stools from medium size dogs (*n* = 2). Lumen and mucus-associated microbiota composition was analyzed by qPCR and 16S metabarcoding. Relative abundance of the main bacterial populations in both colonic microenvironments are represented at the phylum (a), family (b), and genus (c) levels.

Whatever taxonomic levels and colonic microenvironments, profiles obtained with small and medium size conditions were more closely related than large one (Figure 4). At the phylum level (Figure 4a), in the luminal medium, for both donors, we can observe from small to large condition an increase in relative abundances of *Fusobacteriota* (from 22% in small to 33% in large condition), *Firmicutes* (from 14% to 35%) and *Proteobacteria* (from 5% to 28%) whereas *Bacteroidota* decreased (from 59% to 3%). At family level (Figure 4b), *Lactobacillaceae, Coriobacteriaceae*, and *Dialisteraceae* and *Streptococcaceae* (the last one for dog A only) were only present in the large size condition in luminal and mucosal fractions. Moreover, the large size condition presented an important luminal proportion of *Peptostreptococcaceae* (around 5–15%) for dog A and *Enterobacteriaceae* (until 30%) for dog B, balanced by a reduced *Bacteroidaceae* and *Prevotellaceae* proportions (<5%) compared to small and medium groups. Interestingly, some differences observed between donor A and B were kept in the artificial colon, such as higher abundances in *Clostridiaceae*, *Enterobacteriaceae,* and *Lachnospiraceae* in bioreactors inoculated with stool from dog B. At the genus level (Figure 4c), main results obtained at a higher taxonomic level were confirmed with higher relative abundances under large size condition of *Fusobacterium*, *Prevotella*, *Proteus, Peptacetobacter*, *Lactobacillus,* and *Clostridium* (only for dog B), together with lower amounts of *Bacteroides* and *Alloprevotella*.

**Figure 5.**
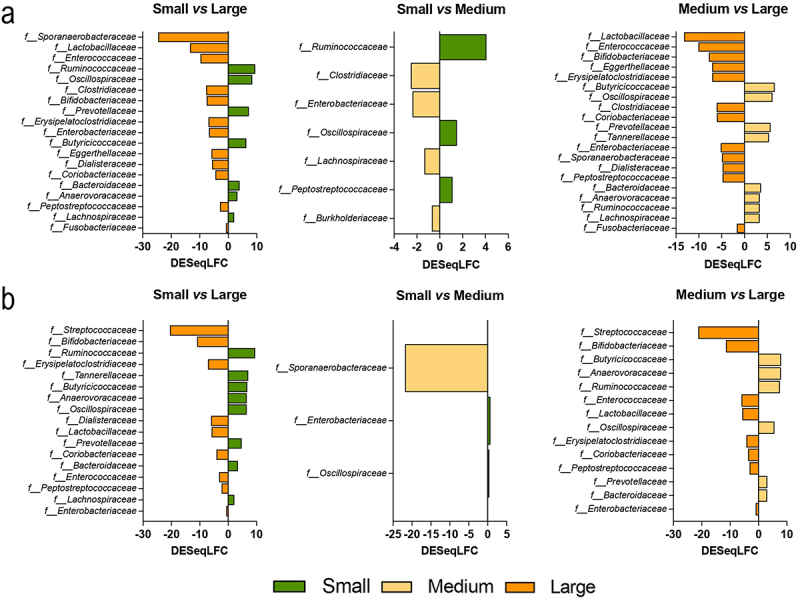
Differential analysis on dog size impact on microbiota composition at the genus level. Fermentations were performed in the CANIM-ARCOL under three dog size conditions (i.e. small, medium, large), after inoculation with stools from medium size dogs (*n* = 2). Lumen and mucus-associated microbiota composition was analyzed by 16S metabarcoding and differential analysis were further performed on days 2 to 9. Differential analyses based on DESeq2 method were performed to generate loading plots of the 10 most contributing genera between sizes in luminal medium (a) and mucin beads (b). Bars are colored according to the group in which the median abundance is maximal, small in green, medium in yellow and large condition in orange.

Discriminant analysis (Figure 5) between the three size groups confirmed that small and medium size conditions were more similar (at the family level) between them than with large size condition (*i.e*. small *vs* large and medium *vs* large). In the luminal compartment (Figure 5a), *Lactobac-illaceae*, *Enterococcaceae*, *Sporanaerobacteraceae*, *Clostridiaceae Peptostreptococcaceae, Coriobacteriaceae, Dialisteraceae, Bifidobacteriaceae,* and *Fusobacteriaceae* were significantly enriched in the large size conditions compared to both medium and small (*p* < 0.05). The most striking differences between small and medium size conditions were the enrichment of *Ruminococcaceae* and *Oscillospiraceae* in small condition, together with an increase in relative abundance of *Clostridiaceae*, *Enterobacteriaceae,* and *Lachnospiraceae* under medium one. In the mucin beads (Figure 5b), some discriminant families of the luminal medium (large *versus* medium and small) are conserved, such as higher relative abundance of *Enterococca-ceae*, *Bifidobacteriaceae, Oscillospiraceae,* and *Lactobacillaceae*. Other families, like *Streptococcaceae*, are highly selective of the large size condition but only in the mucus-associated microbiota.

### Impact of size conditions on canine colonic microbiota activity

**Figure 6.**
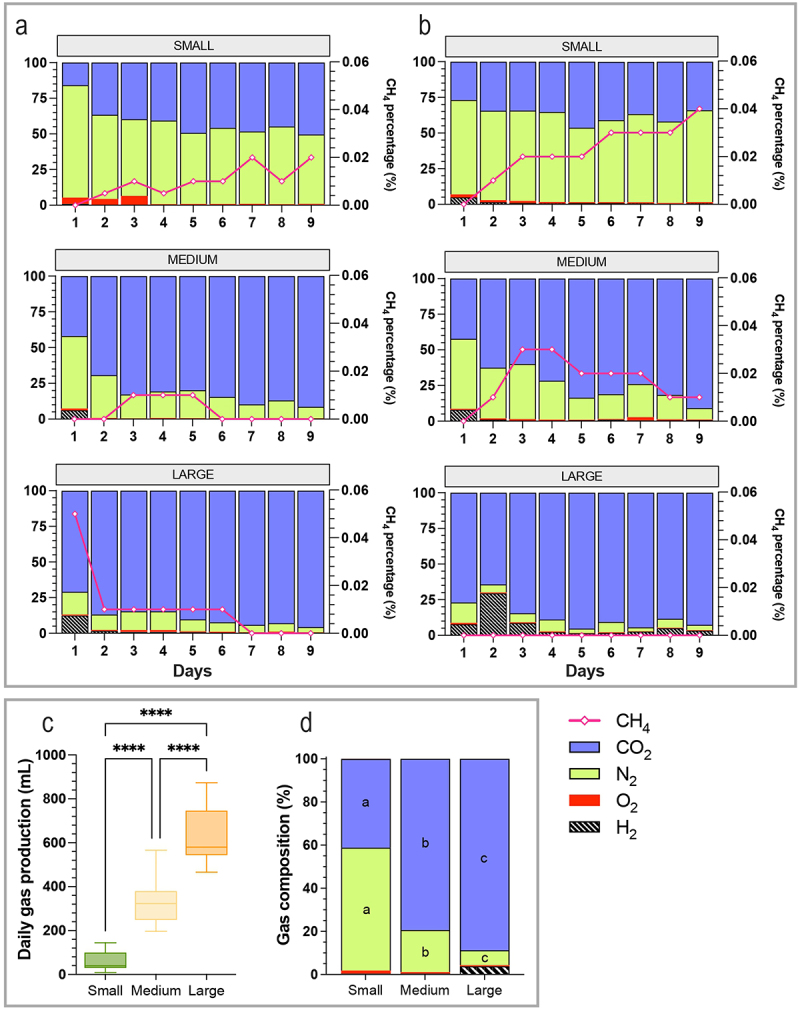
Impact of dog size on gas production in the CANIM-ARCOL. Fermentations were performed in the CANIM-ARCOL under three dog size conditions (i.e. small, medium, large), after inoculation with stools from medium size dogs (*n* = 2). Samples were regularly collected from atmospheric phase of bioreactors to determine gas composition. Results are expressed in relative percentages for dog a (a) and dog B (b). Daily total gas production is given in mL (c). Average gas composition from day 4 to day 9 was calculated per size condition and represented in (d). Statistical differences are indicated by different letters (*p* < 0.05) or ****: *p* < 0.0001 (ANOVA one-way).

Total gas production was evaluated every day and a significant (*p* < 0.0001) increase with size was observed, with medians of 40, 320, and 580 mL per day for small, medium, and large size, respectively (Figure 6c). Gas composition was clearly different depending on size, but for each category quite similar between the two donors (Figures 6a,b). Relative percentages of CO_2_ (Figure 6d) significantly increased with size format (41, 79, and 89% for small, medium, and large size conditions, respectively) as well as H_2_ (0.4, 0.4, and 3.7%). Opposite trends were observed for O_2_, decreasing with dog size from 1.4 for small condition to 0.6% for large one. Both donors were CH_4_-producers with clear impact of size on CH_4_ levels, even if associated percentages remained very low (<1%). The highest percentages were found under small size conditions, with up to 0.04% at the end of fermentations for dog B. In large dog condition, CH_4_ percentages dropped under detection levels for both dogs.

**Figure 7.**
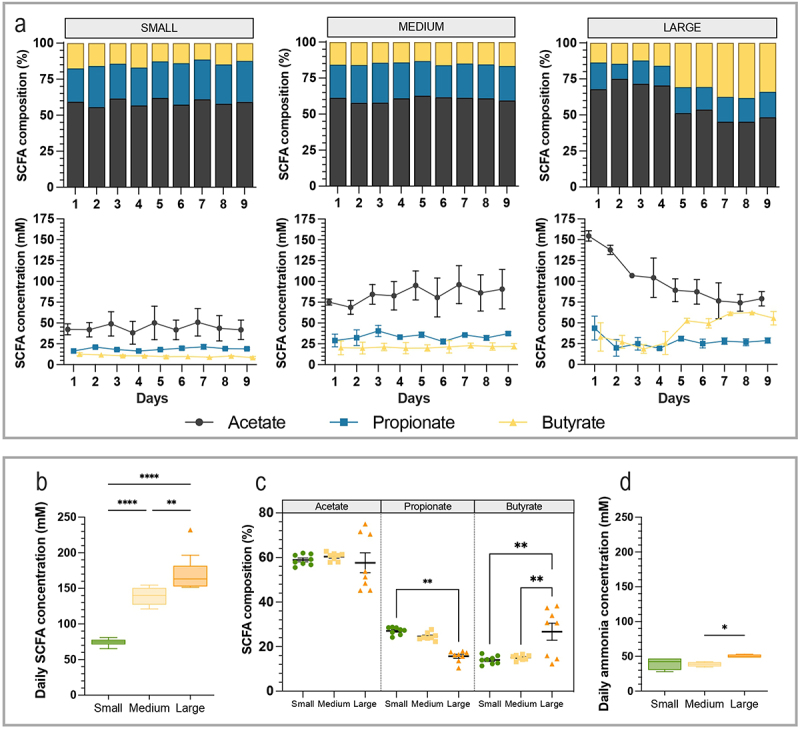
Impact of dog size on short-chain fatty acids and ammonia production. Fermentations were performed in the CANIM-ARCOL under three dog size conditions (i.e. small, medium, large), after inoculation with stools from medium size dogs (*n* = 2). Samples were regularly collected from luminal medium of the bioreactors to determine short-chain fatty acids (SCFA) and ammonia concentrations. The three main SCFA (i.e. acetate, propionate and butyrate) were measured daily throughout fermentations and results expressed in mean relative percentages (a, top) and concentrations (a, bottom). Average SCFA concentrations (b), SCFA composition (c) and ammonia concentrations (d) were calculated per size condition from day 2 to day 9. Statistical differences are indicated by *: *p* < 0.05, **: *p* < 0.01; ****: *p* < 0.0001 (ANOVA one-way).

The three main SCFA (i.e. acetate, propionate, and butyrate) were quantified in the fermentation medium of the bioreactors (Figure 7). Similar profiles of acetate, propionate, and butyrate were observed between small and medium groups with around 60% acetate, 30% propionate, and 10% butyrate (Figure 7a). Bioreactors mimicking large size condition were characterized by around 45–50% acetate, 20% propionate, and an increased proportion of 30–35% butyrate at the end of the experiment. Daily acetate concentrations (average from days 2 to 9, data calculated from Figure 7a) significantly increased from 41.7 mM (small condition) to 94.4 mM (large condition), with differences observed between small and medium (*p* < 0.0001) and small and large conditions (*p* < 0.0001). Daily propionate concentrations did not differ with dog size condition, whereas butyrate increased from 10.5 for small to 44 mM for large with significant differences observed between small and large (*p* < 0.0001) and medium and large (*p* < 0.001) conditions. Daily SCFA production also significantly increased with size, from 75 to 165 mM per day (*p* < 0.001) (Figure 7b). Total SCFA production differences were associated with higher butyrate relative abundance in the large condition bioreactors (Figure 7c).

Lastly, ammonia levels quantified in the luminal medium varied between 40 and 50 mM and were significantly higher in the large size condition than in the medium one (Figure 7d).

### Interactions between explainable variables and family abundances

**Figure 8.**
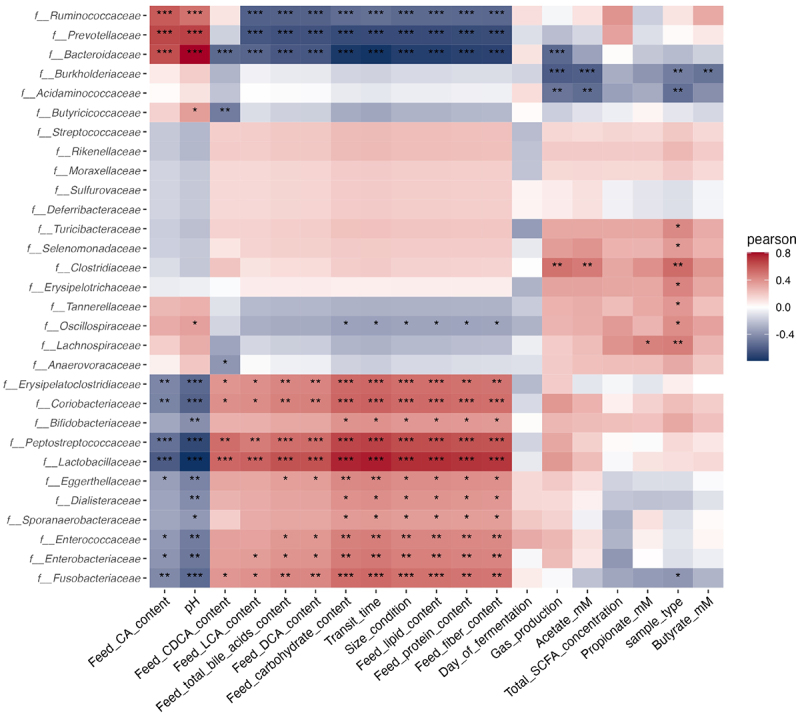
Correlations between explainable variables and microbial family abundances. Fermentations were performed in the CANIM-ARCOL under three dog size conditions (i.e. small, medium, large), after inoculation with stools from medium size dogs (*n* = 2). Lumen and mucus-associated microbiota composition was analyzed by 16S metabarcoding. Explainable variables included day of fermentation, sample type (i.e. luminal medium or mucin beads), physicochemical (i.e. pH, transit time, bile acid profiles) or nutritional (i.e. lipid, protein, carbohydrate and fiber content) parameters of bioreactors, as well as gut microbial metabolites (i.e. gas and SCFA) and bacterial family relative abundances. Luminal medium and mucin beads were combined, and Pearson correlations were calculated on days 2 to 9. Statistical differences are indicated by *: *p* < 0.05, **: *p* < 0.01; ***: *p* < 0.001.

Based on Pearson correlations, we further analyzed our data to try to evidence correlations between day of fermentation, physicochemical (i.e. pH, transit time, bile acid profiles) or nutritional (i.e. lipid, protein, carbohydrate, and fiber content) parameters of bioreactors, as well as gut microbial metabolites (i.e. gas and SCFA) and bacterial family relative abundances (Figure 8). This was performed by combining results from luminal medium and mucin beads, since similar data were obtained with separate analysis (data not shown). Pearson correlations based on the variable ‘size condition’ confirmed previous discriminant bacterial populations between dog sizes (notably *Ruminococcaceae*, *Prevotellaceae*, *Bacteroidaceae*, *Lactobacillaceae*, *Peptostreptococcaceae,* and *Fusobacteriaceae*). We also confirmed the lack of effect of ‘day of fermentation’ variable on bacterial relative abundances, attesting the stability of those populations in the artificial colon. Similar significant correlations were obtained between ‘size condition,’ ‘transit time,’ and all nutritional parameters (‘lipid, protein, carbohydrate, fiber’), suggesting that all those parameters widely patterned bacterial profiles in the bioreactors. Exactly opposite trends were observed with ‘pH,’ indicating that this variable also broadly impacts bacterial population *in vitro*, but this time with a negative correlation with dog size. Regarding bile acids, cholic acid content showed a markedly different correlations with main discriminant populations compared to the three other ones, i.e. chenodeoxycholic, deoxycholic, and lithocholic acids. Besides, even if total SCFA concentration significantly increased with dog size (Figure 7b), Pearson correlations showed no impact of this variable (*p* > 0.05). With regard to each main SCFA, acetate was negatively correlated with relative abundance of *Burkholderiaceae* and *Acidaminococcaceae*, but positively correlated with the one of *Clostridiaceae*. In addition, propionate concentration was significantly linked to an increase in *Lachnospiraceae* relative abundance, whereas butyrate was negatively associated with relative abundance of *Burkholderiaceae*. Lastly, Pearson correlations obtained for total gas production were quite similar to that of acetate concentrations, except for *Bacteroidaceae*.

## Discussion

In a previous study, we developed a new *in vitro* gut model, the CANIM-ARCOL, reproducing the main parameters of the canine colonic ecosystem and adapted to three dog sizes, i.e. small, medium, and large sizes [26]. The model was set up to reproduce the main physicochemical, nutritional, and microbial parameters specific to each dog size and inoculated with fecal samples from size-related conditions (e.g. small size bioreactors were inoculated with stool from small size dogs). In particular, we simulated *in vitro* the increase in colonic transit time, feed nutrients (proteins, carbohydrates, lipids, and fibers), and secondary bile acid concentrations (DCA and LCA) associated with dog size, while pH and primary bile acid (CA) decreased (Table 2), in line with *in vivo* data [10,21,36–41]. After validating this model through *in vivo-in vitro* correlations, we aimed in the present work to provide a mechanistic understanding of how colonic parameters are able to shape microbiota depending on dog size conditions. Of interest, such an *in vitro* approach allows to dissociate the microbial component from nutritional and physicochemical parameters of the colonic ecosystem, which is obviously impossible *in vivo*. Therefore, we inoculated the CANIM-ARCOL with stools from medium-size dogs, while bioreactors were set up to reproduce small, medium, or large colonic conditions. The main objective was then to provide a comprehensive understanding on the relative importance of microbes (based on fecal inoculum) and colonic parameters in modeling microbiota at structural and functional levels, between the three dog sizes.

With this objective in mind, we first performed comparisons on the effect of dog size between the present results and those previously obtained in the CANIM-ARCOL inoculated with fecal samples from small, medium, and large dogs [26], as described in Table 4. We should keep in mind that, even if there are of interest, such comparisons should be hampered by differences between the two studies in time of fermentation (9 days here *versus* 21 days in the previous study) and number of fecal samples (2 dogs *versus* 13 dogs). Impact of dog size on microbial alpha-diversity was in line to previous *in vitro* results, i.e. decreasing with size. However, at the phylum level, we observed opposite tendency or no clear conclusion for the main phyla (*Actinobacteria*, *Bacteroidota*, *Fusobacteriota,* and *Proteobacteria*) in both microenvironments, except for *Firmicutes* in the luminal medium. At the family level, changes in 7 and 5 families out of 12, in the luminal medium and mucin beads, respectively, were in accordance with previous *in vitro* results. However, major families from canine gut microbiota such as *Bacteroidaceae* and *Fusobacteriaceae* demonstrated opposite or unclear size effects between the two studies. Regarding microbiota activity, we evidenced great correlations between the present study and the previous results [26], with 10 out of 11 tested parameters showing similar trends, including total and main SCFAs and gases, as well as ammonia. This suggests functional overlaps between different bacterial taxa resulting in quite consistent microbiota activity. The second level of comparisons to assess the relative importance of microbial and environmental colonic parameters was to compare the present results to *in vivo* data in dogs (Table 5). Due to the paucity of information on canine colonic microbiota *in vivo*, with only two studies in medium dogs [2,22] and none with small and large dogs, *in vitro-in vivo* comparisons were based on fecal data extracted from our literature review (23 studies) [8]. Main families observed in fecal samples of different dog sizes were successfully found *in vitro* (Table 5a), except for *Acidaminococcaceae* (not detected in small and medium dog stools but present under three dog size conditions in the CANIM-ARCOL) and *Veillonellaceae* (previously described in large dog feces but not found *in vitro*). Regarding size effect, we observed similar trends for 6 out of 12 families, no clear conclusion for 4 and opposite trends for *Prevotellaceae* and *Veillonellaceae*. This indicates that physicochemical and nutritional parameters applied *in vitro* were able to shift microbiota according to *in vivo* data for half of the followed families, regardless of the microbial inoculum. Similarly, size effect on microbiota activity was in line with fecal *in vivo* data for three over five studied parameters, including total SCFA and ammonia (Table 5b). One explanation can be that environmental colonic parameters can drive microbiota *in vitro*, especially at the functional level, but are not sufficient to shape bacterial populations with adequate profiles compared to *in vivo* data. This means that inoculating CANIM-ARCOL with fecal samples from three dog sizes remains necessary to obtain relevant *in vitro-in vivo* comparisons.

**Table 4.** *In vitro*–*in vitro* correlations related to dog size effect on colonic microbial populations and metabolic activities between previous results in the CANIM-ARCOL and those obtained in the present study. Previous results were extracted from Deschamps et al. [26] and found when the model was inoculated with fecal samples from three dog sizes. Size effect was indicated by symbols: 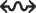 no size effect, 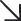 decrease with size or 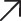 increase with size. Color code indicates similar tendency between the two studies (in green), clear opposite trends (in red) or no clear conclusion (in yellow). *: significant variations between sizes (*p* < 0.05). ND: not detected.

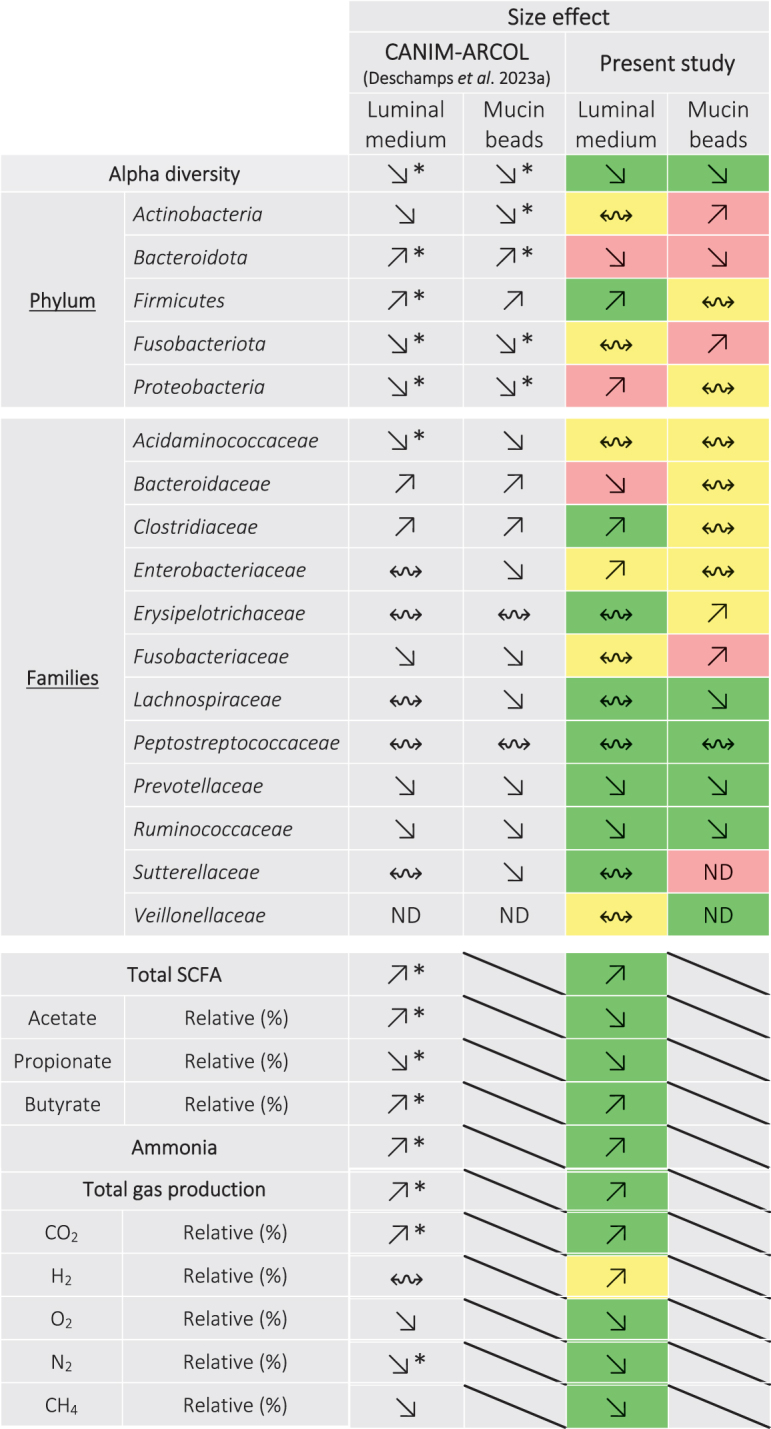

**Table 5.** *In vivo*–*in vitro* correlations related to dog size effect on gut microbial populations (a) and metabolic activities (b) between our *in vitro* results in the CANIM-ARCOL and data from fecal samples *in vivo*. Data from dog fecal samples are extracted from Deschamps et al. (2022). Presence of bacterial populations was indicated by ‘yes’ if present or ‘no’ if absent (not found in the main bacterial populations). Size effect was indicated by symbols: 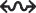 no size effect, 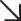 decrease with size or 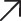 increase with size. Color code indicates similar tendency between *in vitro* and *in vivo* data (in green), clear opposite trends (in red) or no clear conclusion due to lack of data or inconsistencies (in yellow). ND: not determined. [60–67]

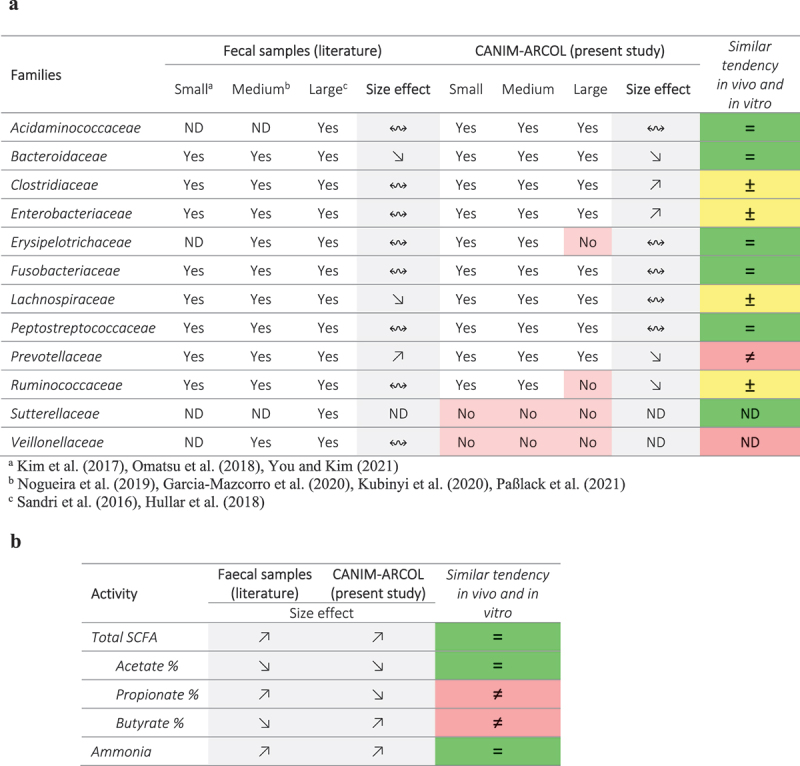

Of interest, this *in vitro* study allowed to better understand how different parameters from the canine colonic ecosystem can drive major bacterial family’s relative abundances (Figure 8). Our results indicated that nutritional and physicochemical parameters related to different dog sizes, but not the main microbial fermentation end-products (i.e. SCFA and gas) are microbiota drivers. In this study, *Coriobacteriaceae* and *Fusobacteriaceae* relative abundances were positively correlated with dog size and protein content (in the simulated ileal effluents), in accordance with *in vivo* studies in medium dogs, where high-protein diet increased the fecal levels of those two bacterial populations [42,43]. Such observations are in line with previous works reporting the involvement of *Fusobacterium* in protein fermentation to produce butyrate [44]. A discriminant enrichment in *Clostridiaceae* and *Lactobacillaceae* was also observed *in vitro* from small to large dog size conditions, associated with higher fiber contents. This is also in adequacy with *in vivo* results showing similar trends in adult medium dog stools when they were fed with a high-fiber diet containing 7.5% beet-pulp [45–47]. Those two families are known to produce SCFA from carbohydrate fermentation in the gut [48], in line with the positive correlation we evidenced in the CANIM-ARCOL between total SCFA concentrations and dog size. In addition to feeding, bile acids are also acknowledged as key factors shaping the intestinal microbiota composition [49]. In the CANIM-ARCOL model [26], primary bile salt content in the ileal effluents decreased with dog size, while secondary bile salts increased, extrapolated from fecal *in vivo* data showing different bile acid profiles and total bile acid content in feces from small, medium, and large dog sizes [21]. Changes in bile salt concentrations depending on dog sizes were primarily correlated with modifications in *Bacteroidaceae*, *Lactobacillaceae*, *Clostridiaceae,* and *Ruminococcaceae* abundances, in accordance with the main families involved in bile salt metabolism, through dihydroxylation, oxidation, or epimerization [21,49,50].

Besides nutrients and bile salts, we also showed that physicochemical parameters of the canine colonic ecosystem are key factors shaping microbiota *in vitro*. In particular, negative correlations between transit time and *Bacteroidaceae* relative abundances were evidenced in the CANIM-ARCOL. Once again, this result should be linked to *in vivo* data since *Bacteroidaceae* raised in stools from dogs with chronic diarrhea, showing a reduced transit time [51]. Faster transit time can impact nutrient supply in the gut (leading to excess of resources) and select species from *Bacteroidaceae* able to grow rapidly during reduced competition [52]. In addition, we observed in the present work a negative correlation between bacterial alpha-diversity and dog size, meaning that diversity decreased with transit time. Our results are in line with a previous study in an *in vitro* model of the human large intestine showing that a longer transit time (associated with aging in human) led to a drop in microbial diversity and an increase in *Clostridiaceae*, *Enterobacteriaceae,* and *Coriobacteriaceae* abundances and total SCFAs [53]. Moreover, increasing transit time in the CANIM-ARCOL induced enrichment in *Lactobacillaceae*, *Enterococcaceae,* and *Bifidobacteriaceae*, in full accordance with a previous work in an *in vitro* human gut model showing that an increased transit time (from 5 to 10 h *versus* 5 to 15 h in the present study) was associated with a rise in *Lactobacillus*, *Enterococcus,* and *Bifidobacterium* [54]. These families are involved in carbohydrate fermentation and lactate production, which can be promoted by increased transit time allowing a longer contact between bacteria and macronutrients [54]. Lastly, transit time was negatively correlated with CH_4_ percentages. CH_4_ is primarily produced by methanogenic *Archaea*, poorly described up to now in dog stools *Archaea* [47], but previously recovered in the CANIM-ARCOL (and identified as *Methanobrevibacter smithii*), especially under small size conditions [26]. A slow transit time was previously described as facilitating factor for *Archaea* development and associated with an increased methane production in human [55], which is contradictory with our results and suggests that in addition to transit time, other parameters such as availability of nutrients can favor CH_4_ production. The other physicochemical parameters driving microbiota in our study are colonic pH. In the CANIM-ARCOL, values decreased from 6.5 to 6.2 from small to large size conditions, associated with a decrease in propionate but an increase in butyrate concentrations. pH was positively correlated with *Bacteroidaceae*, while *Actinobacteria* and *Firmicutes* decreased. All those results are in perfect line with those found by Haindl *et al*. [56] *in vitro* in human feces. However, these authors also observed an increase in total SCFA concentrations when pH increased (from 6 to 7), while opposite results were obtained here, certainly due to differences in nutrient availability.

To conclude, this study provides for the first time relevant preliminary mechanistic insights about the relative importance of gut microbes and colonic physicochemical and nutritional parameters in modeling canine microbiota by using the CANIM-ARCOL. We showed that environmental colonic parameters (such as nutrient availability, transit time, or pH) seem to be sufficient to drive microbiota at the functional level, but that size-related fecal microbes were necessary to accurately reproduce the colonic environment of small, medium, and large dogs. This *in vitro* study also allowed to evidence main bacterial populations shaped by nutritional or physicochemical parameters in the canine colonic ecosystem. Further investigations would be necessary to confirm the relative importance of each of those parameters in shaping gut microbes. Especially, the recent developments of noninvasive methods to monitor digestive parameters, such as pH, motility, or transit time (wireless motility capsules, e.g. SmartPill) in dogs [68], together with new methods allowing a site-specific sampling of the gut microbiome [57] may help to better understand the relative impact of such parameters in modeling the canine microbiota. In a next future, the CANIM-ARCOL model can be used as a relevant *in vitro* tool to decipher the relative importance of microbiota *versus* environmental colonic parameters in food and pharma studies, e.g. when studying nutrient/drug bio-accessibility or probiotic/enteric pathogen survival and activity. This study was focused on the effect of dog sizes, but the potential of our model can be extended to the simulation of different ages or healthy *versus* diseased situations (e.g. chronic enteropathies or obesity), helping to move toward personalized dietary of medicine recommendations for dogs [58,59].

## Data Availability

Raw metabarcoding data that support the findings of this study are openly available at https://www.ncbi.nlm.nih.gov, BioProject number PRJNA955438.
